# Georgia Medical Association

**Published:** 1873-04

**Authors:** 


### PART III.

### EDITORIAL AND MISCELLANEOUS.

*Georgia Medical Association.*

This body held its annual meeting in Atlanta on the 9th instant. Many valuable papers were read, which will make the volume of Transactions one of great interest. The paper of Dr. Robert Battey, in defense of his operation of Normal Ovariotomy, was one of unusual merit for its fidelity to truth, closeness of reasoning, and thoroughness of research. Our space and want of time forbids our saying more at present, but we will notice the proceedings of the Association at greater length when they shall be published. The Association meets on the first Wednesday of April, 1874, at Thomasville, Georgia.

